# The ENACT network is acting on housing instability and the unhoused using the open health natural language processing toolkit

**DOI:** 10.1017/cts.2024.543

**Published:** 2024-05-16

**Authors:** Daniel R. Harris, Sunyang Fu, Andrew Wen, Alexandria Corbeau, Darren Henderson, Jordan Hilsman, David Oniani, Yanshan Wang

**Affiliations:** 1 Center for Clinical and Translational Sciences, University of Kentucky, Lexington, KY, USA; 2 Institute for Biomedical Informatics, University of Kentucky, Lexington, KY, USA; 3 Center for Translational AI Excellence and Applications in Medicine, University of Texas Health Science Center at Houston, Houston, TX, USA; 4 Department of Health Information Management, University of Pittsburgh, Pittsburgh, PA, USA; 5 Clinical and Translational Science Institute, University of Pittsburgh, Pittsburgh, PA, USA; 6 Department of Biomedical Informatics, University of Pittsburgh, Pittsburgh, PA, USA

**Keywords:** Housing instability, natural language processing, social determinants of health

## Abstract

Housing is an environmental social determinant of health that is linked to mortality and clinical outcomes. We developed a lexicon of housing-related concepts and rule-based natural language processing methods for identifying these housing-related concepts within clinical text. We piloted our methods on several test cohorts: a synthetic cohort generated by ChatGPT for initial infrastructure testing, a cohort with substance use disorders (SUD), and a cohort diagnosed with problems related to housing and economic circumstances (HEC). Our methods successfully identified housing concepts in our ChatGPT notes (recall = 1.0, precision = 1.0), our SUD population (recall = 0.9798, precision = 0.9898), and our HEC population (recall = N/A, precision = 0.9160).

## Introduction

Environmental social determinants of health (SDOH), such as one’s living circumstances and housing stability, significantly impact a person’s overall health, and the lack of stable housing can lead to serious adverse effects [1]. Housing directly impacts a person’s access and means to medical care; lack of housing is related to a disproportionately higher reliance on emergency medical services and ambulance transports [2]. The most severe manifestation of housing instability, known as housing deprivation or homelessness, can reduce life expectancy by as much as 12 years and increase rates of illness or disability [3,4]. Experiencing homelessness is linked to notably increased rates of hospital readmissions and extended hospital stays [5].

Despite the importance of housing and its relevance to health, housing issues are underreported in electronic health records (EHRs) due to a lack of national standards, social stigma, and reliance on self-reporting [6]. A previous study on housing found that diagnosis codes used for billing only identified 58.5% of the population experiencing housing instability or homelessness [7]; the remaining population was only identifiable through clinical notes or address data [7]. Clinical text combined with natural language processing (NLP) techniques may assist in identifying housing issues from unstructured data in EHRs [8–12]. We extend these housing-related techniques and findings as part of a national effort to capture housing-related concepts.

The Evolve to Next-Gen Accrual to Clinical Trials (ENACT) Network spans the Clinical and Translational Science Award (CTSA) consortium and connects CTSA sites with a single interface capable of querying over 142 million patients using the ENACT web-based query tool [13]. One of the goals of ENACT is to allow informatics researchers to develop and validate new EHR research tools; a working group (WG) for developing NLP tools was established across participating ENACT sites. This paper outlines the WG’s progress on using clinical text to help identify housing issues and to supplement the known gap of underreported housing instability in structured clinical data by using NLP with unstructured EHR data. We present our custom lexicon of housing-related terms constructed after a literature review and discuss the performance of our initial implementation using three unique data sets.

## Materials and methods

### Lexicon development

We conducted a literature review of studies involving housing instability and homelessness to identify relevant works and to help construct a lexicon of housing-related terms [7,12,14,15]. An existing Open Health Natural Language Processing (OHNLP) project on food and housing insecurity was reviewed to compare important words, phrases, and patterns [16]. We organized our relevant findings into six concepts: homeless, unstable housing, recovery housing, emergency housing, temporary housing, and exposure. These concepts and associated phrases are summarized in Table 1 and were selected to support fine-grain querying of housing in the ENACT query tool and clinical trial recruitment.

**Table 1.** Housing-related concepts and phrases

Concept	Keywords or phrases
Homeless	Houseless, homeless, houselessness, homelessness, unhoused, unsheltered, unsheltered homelessness, lack of housing, lack of shelter, lacking housing, lacking shelter, housing lack, homelesss
Unstable housing	Vulnerably housed, unstably housed, unstable housed, unstable housing
Recovery housing	Recovery housing, recovery residence
Emergency housing	Emergency housing, emergency shelter
Temporary housing	Transitional housing, temporarily staying, itinerant, shelter, couch surfing, lodgers
Exposure	Evicted, living on the streets, residential institution problems, sleeping outside, sleeping outdoors

### Algorithm development

We developed patterns to identify housing-related issues that were compatible with the OHNLP Toolkit, developed by the OHNLP consortium, for automated concept extraction from clinical notes [17]. The OHNLP Toolkit was selected due to its customizable interfaces which could support NLP efforts in multiple domains, including those beyond housing, and its easy integration with the ENACT query tool. This toolkit utilizes MedTagger, a lightweight tool for indexing based on dictionaries and patterns as the core component for information extraction [17,18]. The phrases in Table 1 were converted to regular expressions, which compressed the list. For example, “lack of housing” and “lack of shelter” are reduced to one pattern: “lack of (shelter|housing).” Furthermore, patterns were developed to allow flexible matching. For example, “living on the streets” became “living on the (the)? street(s),” where the article “the” is optional and streets may be plural or not. A common misspelling of “homeless” as “homelesss” was added based on the observational experience of the team in the housing domain.

The OHNLP Toolkit uses an expanded version of the ConText algorithm to classify whether identified entities are negated or part of a patient’s medical history [19]. Irrelevant ConText rules, such as “did not demonstrate,” were removed from the rule list to avoid wrongly negating detected entities; housing issues are not items for which patients test positive or negative. Each clinical document was divided into sentences as a preprocessing step; this was necessary after observing hits with negations generated from the wrong contextual window. These sentences were input into the OHNLP Toolkit, and annotated text files of the results were produced as output.

### Testing

Each participating implementation site developed its own test data. For piloting the implementation, we developed a collection of emergency department notes using ChatGPT 3.5 that could be shared across sites for testing purposes. The initial question was “Can you write a sample discharge note from an Emergency Department for a homeless person?” Several additional prompts were used to generate positive cases in which the hypothetical patient has housing problems and negative cases in which there is no housing concern (“Can you generate a report for someone who is not homeless and not experiencing housing instability?”).

We repurposed a selection of 250 documents from a related study on housing within a cohort with substance use disorders (SUD) specific to stimulant and opioid use disorders (randomly sampled from individuals having ICD-10-CM diagnosis codes of F11.*, F14.*, F15.*, T40.[1-6].*, and T43.6*) [7]. These documents were manually annotated as positive or negative for housing issues; patients experiencing housing issues have higher rates of SUD and are at higher risk of overdose, which highlights the importance of housing as an SDOH [20]. We also created a collection of 24,917 documents from a cohort (*n* = 225) diagnosed (ICD-10-CM Z59.6) with problems related to housing and economic circumstances (HEC) from UT Physicians, a multispecialty medical group associated with the University of Texas Health Sciences Center at Houston (UTHealth) and the UTHealth Harris County Psychiatric Center.

## Results

The results of running the OHNLP Toolkit with our custom ENACT rule set and custom patterns on our three test data sets are summarized in Table 2. The results of MedTagger contain a flag for negation per each hit; a note was considered a positive case for housing issues if any of its hits were positive. True negatives were cases that either had no documented housing issues or all mentions of housing were negated. For the HEC cohort, the extracted hits were reviewed for correctness, so only precision is reported.

**Table 2.** Performance using ENACT NLP rules

Cohort		Present (actual)	Absent (actual)	Recall	Precision
ChatGPT	**Present (rules)**	25 (TP)	0 (FP)	1.0	1.0
	**Absent (rules)**	0 (FN)	10 (TN)		
SUD	**Present (rules)**	194 (TP)	4 (FP)	0.9798	0.9898
	**Absent (rules)**	2 (FN)	50 (TN)		
HEC	**Present (rules)**	403 (TP)	37 (FP)	N/A	0.9159
	**Absent (rules)**	N/A	N/A		

ENACT = Evolve to Next-Gen Accrual to Clinical Trials; FN = false negative; FP = false positive; HEC = housing and economic circumstances; NLP = natural language processing; SUD = substance use disorders; TN = true negative; TP = true positive.

Table 3 lists common errors observed. For the SUD collection, false positives mostly stemmed from “Patient Education” notes that list dozens of community resources available for any patient; the “Homeless Veterans Center” caused false positives as it was only listed as a generic resource and did not imply the patient was a homeless veteran. Another false positive stemmed from a note describing someone who visited the emergency department after finding “a homeless person sleeping in her bathroom.”

**Table 3.** Examples of errors

Cohort	Error	Sentence
SUD	False positive	“… found a **homeless** person sleeping in her bathroom.”
	False positive	“**Homeless** Veterans Center” (listed in a directory of community resources, but this patient had no documentation of experiencing housing issues.)
SUD	False negative	“He does **not** want to return to the community homeless shelter where he was living, and has no money, transportation or job.”
	False negative	“Patient is **not** safe candidate for home IV abx therapy given active IVDA and homelessness.”
	False negative	“He does **not** want to return to his previous living situation due to the drug use in the home but also refuses to go to a homeless shelter and has nowhere else to go.”
	False negative	“Patient **not** reveal to staff that she was homeless, but per (transferred hospital) patient stated she was homeless.”
	False negative	“Pt **not** agreeable to homeless shelter and requested Ronald McDonald House.”
	False negative	“Patient reports he presents because he believes that he needs mental health services related to frequent “outbursts” which have gotten him in trouble in the past, and resulted in him **not** being able to stay with his father or mother, and thus being homeless.”
	False negative	“Patient is **not** safe candidate for home IV abx therapy given active IVDA and homelessness – will need to be kept in monitored environment for duration of IV antibiotics.”
	False negative	“Regarding TB risk factors – the patient was homeless for 4 months recently, but did **not** stay in a homeless shelter, he has never worked in healthcare, he was in jail in the 1980s.”
HEC	False positive	“Son was homeless and patient was sending food.”
HEC	False positive	“Unstable Housing in the Last Year: No”
HEC	False positive	“They do not remove people from the home in effort to avoid adding to the homeless population.”

HEC = housing and economic circumstances; SUD = substance use disorders.

For the SUD cohort, there were only 2 false negatives at the note level, where all individual hits were negated, and at the individual hit level, there were 10 false negatives; these hits are described in Table 3. These examples are all failures to understand what concept is being negated in the sentence. For example, in “*Patient is not safe candidate for home IV abx therapy given active IVDA and homelessness,*” the concept of a safe candidate is intended to be negated instead of homelessness.

We report the distribution of concepts identified for each cohort in Table 4. The most frequently identified concept across all cohorts was homelessness. The second most frequent concept varied across cohorts. Temporary housing was likely popular in the SUD cohort due to a large number of patients staying in shelters; the HEC cohort was a general population where unstable housing may be more common than staying in a shelter.

**Table 4.** Distribution of concepts per data set

	Identified concept count		
Concept	ChatGPT	SUD	HEC
Exposure	4 (4.4%)	2 (0.5%)	8 (1.8%)
Homeless	50 (54.9%)	333 (84.3%)	373 (84.8%)
Housing instability	26 (28.6%)	0 (0.0%)	3 (0.7%)
Temporary housing	5 (5.5%)	57 (14.4%)	2 (0.5%)
Unstable housing	6 (6.6%)	3 (0.75%)	54 (12.2%)
Grand total	91	395	440

HEC = housing and economic circumstances; SUD = substance use disorders.

## Discussion

Our pilot suggests that developing a lexicon for housing-related issues and rule-based NLP methods for identifying housing concepts in unstructured EHR data is a realistic goal for the ENACT Network. The OHNLP platform is easily deployable and customizable by any ENACT site. The OHNLP toolkit can be customized to read and write to any database; the input can be clinical data warehouses containing the clinical notes and the output can be the ENACT database that stores the searchable patient observations.

The ENACT web-based query tool is based on a browsable ontology that organizes concepts and codes that can be used in a “drag and drop” fashion. Our housing results are searchable on two tiers: the overall housing concept and the embedded individual concepts described in Table 1.

Our ChatGPT performance was without error; this performance is largely unrealistic and likely a reflection of how formulaic ChatGPT output appears. Additionally, ChatGPT occasionally documented negative cases of housing issues as “no visible signs of homelessness” which is highly unlikely to occur in a real note; if a patient does not appear homeless, the clinical documentation for homelessness would simply be absent. The phrase “no visible signs of homelessness” may be pejorative if included in clinical documentation. Despite these limitations, the ChatGPT notes are useful for prototyping and setting up the infrastructure needed to run MedTagger and to interface with the ENACT Network. We leave improving ChatGPT’s formulaic responses as future work where prompt engineering could potentially produce a more realistic data set. We leave exploring the role of generative models in identifying housing issues as future work.

Our SUD results highlighted a false positive where the note references an unhoused individual who is not the patient; this example would be difficult to fix using rule-based methods as there are very little contextual clues or markers in these sentences to emphasize the unhoused person was not the patient. The HEC results highlighted an example where the patient’s family member was experiencing homelessness, which may be addressable by fine-tuning the ConText algorithm to correctly identify family history.

Our study is limited by the breadth and depth of our housing lexicon. Although our intent was to be comprehensive, there may exist phrases or patterns that were not found during our literature review or during our tests. Furthermore, the language used to describe patients experiencing housing problems may change over time. We did not study recall in the HEC cohort due to the large number of notes; a smaller sampling strategy may be needed to manually review and validate recall. We also did not evaluate the temporality of the housing concepts or occurrences of where stable housing is explicitly mentioned.

## Conclusion

The ENACT Network is based largely on querying structured, standardized codes; diagnostic billing codes are insufficient for identifying patients experiencing housing instability or homelessness. We designed our housing lexicon and rule-based NLP methods based on a literature review of other studies and how they reference housing issues. We piloted our methods across a small group of ENACT sites and will be moving to implement these findings as routine updates to the entire ENACT Network, where cohort size estimates can be calculated across sites and in support of innovation clinical trials involving those experiencing housing instability.
